# Cell Monitoring and Manipulation Systems (CMMSs) based on Glass Cell-Culture Chips (GC^3^s)

**DOI:** 10.3390/mi7070106

**Published:** 2016-06-24

**Authors:** Sebastian M. Buehler, Marco Stubbe, Sebastian M. Bonk, Matthias Nissen, Kanokkan Titipornpun, Ernst-Dieter Klinkenberg, Werner Baumann, Jan Gimsa

**Affiliations:** 1 Leibniz Institute for Farm Animal Biology, Institute of Muscle Biology and Growth, Wilhelm-Stahl-Allee 2, 18196 Dummerstorf, Germany; buehler@fbn-dummerstorf.de; 2 Department of Biology, University of Rostock, Gertrudenstr. 11a, 18057 Rostock, Germany; marco.stubbe@uni-rostock.de (M.S.); sebastian.bonk@uni-rostock.de (S.M.B.); Matthias.Nissen@gmx.de (M.N.); werner.baumann@uni-rostock.de (W.B.); 3 Faculty of Science and Technology, Department of Physics, Suratthani Rajabhat University, Surat Thani 84100, Thailand; kmaswiwat@hotmail.com; 4 DOT GmbH, Charles-Darwin-Ring 1A, 18059 Rostock, Germany; klinkenberg@dot-coating.de

**Keywords:** sensorized cell-culture chip, microfluidics, AC-electrokinetics, drug screening, multi-electrode array, interdigitated electrode structure, Clark-type oxygen electrode, potentiometric pH sensor, dielectrophoresis, electroporation

## Abstract

We developed different types of glass cell-culture chips (GC^3^s) for culturing cells for microscopic observation in open media-containing troughs or in microfluidic structures. Platinum sensor and manipulation structures were used to monitor physiological parameters and to allocate and permeabilize cells. Electro-thermal micro pumps distributed chemical compounds in the microfluidic systems. The integrated temperature sensors showed a linear, Pt1000-like behavior. Cell adhesion and proliferation were monitored using interdigitated electrode structures (IDESs). The cell-doubling times of primary murine embryonic neuronal cells (PNCs) were determined based on the IDES capacitance-peak shifts. The electrical activity of PNC networks was detected using multi-electrode arrays (MEAs). During seeding, the cells were dielectrophoretically allocated to individual MEAs to improve network structures. MEA pads with diameters of 15, 20, 25, and 35 µm were tested. After 3 weeks, the magnitudes of the determined action potentials were highest for pads of 25 µm in diameter and did not differ when the inter-pad distances were 100 or 170 µm. Using 25-µm diameter circular oxygen electrodes, the signal currents in the cell-culture media were found to range from approximately −0.08 nA (0% O_2_) to −2.35 nA (21% O_2_). It was observed that 60-nm thick silicon nitride-sensor layers were stable potentiometric pH sensors under cell-culture conditions for periods of days. Their sensitivity between pH 5 and 9 was as high as 45 mV per pH step. We concluded that sensorized GC^3^s are potential animal replacement systems for purposes such as toxicity pre-screening. For example, the effect of mefloquine, a medication used to treat malaria, on the electrical activity of neuronal cells was determined in this study using a GC^3^ system.

## 1. Introduction

New cell-culture techniques are needed to allow cost-efficient, high-content screening of physiologically active and toxic substances in basic research and to replace test animals. Many established cellular assays for determining characteristic cellular parameters (i.e., concentrations of mRNA, proteins, metabolic products, or rates of cell migration, proliferation, and other processes) employ luminescent, fluorescent or radioactive markers. However, the detection principles of the techniques interfere with the detected parameters or with cell physiological aspects, e.g., protein conformation and function, and they may inhibit receptors or disrupt metabolic pathways. In many types of assays, the cells must be lysed to allow the markers to bind to their targets. Accordingly, the assays provide snapshots or "end points" but do not reveal how the parameters evolved under the influence of the tested substances.

Lab-on-chip systems offer a new option for the non-invasive, online determination of the physiological, metabolic and behavioral properties of cells. In such systems, the analytical and cell-culture components are combined in compact systems known as cell monitoring and manipulation systems (CMMSs) [1,2,3,4,5,6,7,8,9,10,11]. This new category of bioanalytical lab-on-chip system allows the online detection of changes in the physiological parameters of respiration, acidification, adhesion and electrophysiological activity of single cells and cellular networks depending on the concentration of the tested compound [4,5,6,9,10,11,12,13,14,15]. CMMSs can thus be useful tools for identifying and characterizing substances that pose a hazard to animals and humans.

International health organizations are increasingly concerned about substances that are potentially neurotoxic and developmentally neurotoxic [16,17]. For example, the new guidelines of the United States Environmental Protection Agency (U.S. EPA) and the Organization for Economic Cooperation and Development (OECD) require testing the developmentally neurotoxic potential of pesticides [18,19]. A drastically increased number of animal experiments will be a direct consequence of such guidelines if conventional methods are used to conduct these tests. We believe that using CMMSs to pre-screen potentially harmful chemicals will reduce the number of sacrificed animals.

The first CMMSs were developed in the 1970s using silicon chip technology. This technology allowed integrating a number of different types of sensors, such as sensors to evaluate cellular respiration, acidification, adhesion, motility and electrophysiological activity [4,5,6,7,8,9,10,11]. Some of these systems had microfluidic pumps and valves integrated into the electronic periphery of the microchips. Such systems allowed bioanalysis, cell manipulation and DNA separation. Commercial CMMSs use silicon-chip or glass-chip technologies (such as the Bionas Discovery^®^ 2500 analyzing system (Bionas GmbH, Rostock, Germany) and the MEA2100-System (Multi Channel Systems, Reutlingen, Germany)) [1,2,20]. The current focus of the field is improving the cell-culture properties of such systems and their capacities for biochemical analysis and clinical diagnostic applications, such as conducting polymerase-chain reactions and patch clamping [3,21,22]. Commercial glass-based systems in the 96 well-plate format, such as the Bionas Discovery^®^ adcon Reader (Bionas GmbH, Rostock, Germany) and the Roche xCELLigence System (Roche Life Science, Barcelona, Spain), allow detecting changes in cell adhesion over seconds or days. However, these systems do not include sensors for determining other physiological parameters, i.e., those involved in respiration or in acidification of media. The Bionas Discovery^®^ 2500 analyzing system allows the parallel, on-line determination of three cell-metabolic parameters (respiration, acidification and adhesion) using six parallel chips over hours and for as long as days. Nevertheless, six chips with different concentrations of substances are insufficient to cover the concentration range necessary to determine their IC_50_ values without first conducting range-finding experiments [13]. Analytical technologies have been combined with cell manipulation technologies in some systems [23,24,25,26,27,28]. Examples of such systems are the automated patch-clamp systems Port-a-Patch^®^, Patchliner^®^ and SyncroPatch (all produced by Nanion Technologies, Munich, Germany), in which an analytical microfluidic chip is used to consecutively study single suspended cells [29,30,31]. The silicon chip includes a glass-pipette that mimics the electrical properties of a conventional patch-clamp electrode. Other high-content screening systems that are based on silicon chip technology are the Cytocon 400™, Cytomen™, and CytoClone™ systems (all produced by Evotec Technologies, Hamburg, Germany) and the Cenix Oncology MultiPlex™ system (Cenix Biosciences, Dresden, Germany) [23,24].

Because the electrical and optical properties as well as the biocompatibility of glass are superior to those of silicon, glass chips have been developed for the extracellular detection of electrical activity, e.g., the action potentials of neuronal cells. In the early 1970s, microelectrode arrays (MEAs) were produced on glass wafers that included a cell-culture trough made using a simple rubber gasket. The groups of G. Gross and J. Pine [20,32,33] achieved a breakthrough in MEA technology, which was then applied in pharmacological surveys. Spontaneously active in vitro neuronal networks located on MEAs can be used to determine substance- and concentration-dependent neurotoxic effects [34]. The aim of current approaches is substance screening by "neuronal fingerprinting" substances, which is determining specific substance-mediated alterations in the activity patterns of the networks [10,35,36].

The aim of our study was to develop GC^3^ systems with integrated glass-based sensors and actuators that are robust, autoclavable, reusable and do not prevent the microscopic observation of the cultured cells. GC^3^ systems that combine these components with certain microfluidic devices can be produced according to the subject of investigation, thereby offering new possibilities for investigating the effects of chemical substances on cellular physiology and metabolism.

For example, we created a glass neurochip (GNC) that combined a multi-electrode array (MEA) with an interdigitated electrode structure (IDES), a temperature probe, and stimulation and ground electrodes. The efficacy of the GNC in detecting the cytotoxic and neurotoxic effects of the anti-epileptic drug valproate [10] and the gap-junction blocker mefloquine on murine embryonic primary neuronal cells (PNC) was tested. Another chip combined metabolic oxygen and pH sensors with an IDES. Whereas the metabolic sensors allowed monitoring of the oxygen consumption and medium-acidification rates of cells, IDES-impedance detection could be used for on-line monitoring of cell spreading, growth, proliferation and migration, which are sensitive indicators of the physiological state of cells [4,5,6,37,38,39,40].

Electro-thermal micro pumps (ETµPs) are ideal tools for overcoming the diffusion limit and distributing media and chemical compounds on GC^3^s. Thermal gradients and high-frequency electric fields can be used to operate the pumps [41,42,43,44]. These pumps do not require moving parts or electrolytic contact between the electrodes and the cell culture media, in contrast to electroosmotic pumps [5].

Whereas AC fields can be used to manipulate culture media, inhomogeneous AC fields can be used to sort and manipulate suspended cells and to allocate them to certain positions on a GC^3^ using dielectrophoresis (DEP) [5]. The DEP-mediated allocation of neurons to MEA electrodes can increase the probability of detecting signals from neuronal networks [28,35].

A third application of AC fields is cell electroporation, which involves the dielectric degradation of the cytoplasmic membranes by high-field pulses [45]. Depending on the parameters of the poration pulse delivered, electroporation can lead to cellular disintegration as a first step in single cell genetic analyses or to transient electro-permeabilization to introduce foreign molecules that do not normally penetrate cellular membranes, such as DNA. Recent medical applications of electroporation include electro-chemotherapy of cancer, gene therapy of somatic cells, and transdermal drug delivery [46,47,48]. When applied to single cells, electroporation can be utilized for cell transfection or for cell hybridization for the production of monoclonal antibodies [49].

## 2. Sensor and Manipulation Structures

### 2.1. Design and Fabrication of the Structures

All of the sensors and passivation structures were designed using Auto-CAD^®^ 2010 software (Autodesk GmbH, Munich, Germany). Before manufacture, the functionality of the sensors was evaluated through finite-element simulations of the electrical, thermal, and fluidic-flow field distributions using COMSOL Multiphysics^®^ software (Comsol Multiphysics GmbH, Göttingen, Germany).

**Figure 1:**
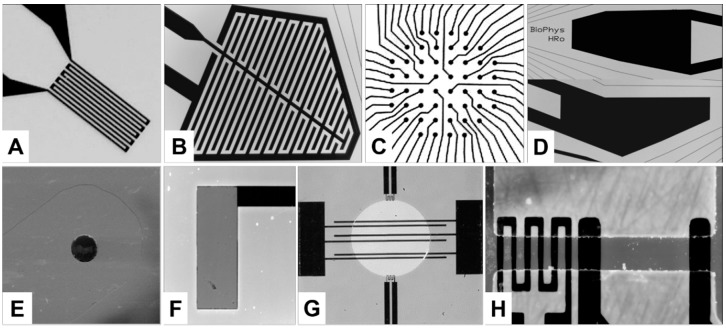
Images of glass-based Pt sensors and actuators (for scaling and the arrangement on two different chip surfaces, see Figure 2). The structures were produced by sputtering glass wafers with 100-nm thick Pt layers. (**A**) temperature sensor; (**B**) IDES; (**C**) MEA; (**D**) two ground electrodes, which can also be used to stimulate cells; (**E**) scanning electron microscopy (SEM) image of a bare oxygen-sensor spot (diameter: 25 µm); (**F**) pH sensor with a rectangular pH-sensitive 1.24 mm × 0.46 mm area (insulated connector is black); (**G**) electroporation chip with a circular test volume and six electroporation electrodes of 80 µm in width and 4.65 mm in length, with electrode distances of 80, 100, 150, 300, and 450 µm, and temperature sensors at the top and the bottom; (**H**) ETµP with a 120-µm wide horizontal microfluidic channel. The black Pt structures form a heating meander with 30-µm wide intervals as well as two 100-µm wide field electrodes separated by 390 µm.

The appropriate platinum (Pt) structures (thickness: 100 nm) were photolithographically produced on 1.1-mm or 0.55-mm thick 4” glass wafers (D236T thin glass, Schott AG, Mainz, Germany). The structures were produced via thin-layer technology using ion beam-processed chrome masks (GeSiM mbH, Grosserkmannsdorf, Germany). With the exception of the sensor and contact areas, the IDES, MEA, oxygen electrode, and contact pad structures were passivated by applying a 1-µm thick layer of silicon nitride (Si_3_N_4_). In the case of the pH sensors, a 60-nm thick pH-sensitive Si_3_N_4_ layer was deposited on the Pt structure. Figure 1 provides an overview of sensor and actuator structures, which are discussed below.

### 2.2. Temperature Sensors

The geometries of the temperature sensors were designed to match the properties of the Pt1000 (Figure 1A) or Pt100 (Figure 1G) industrial standard. To characterize the temperature sensors, we used the thermo-control system of our microscopic stage to heat the water-filled chip troughs. An PT-104A thermometer (Omega Engineering Inc., Stamford, CT, USA) was used to determine the temperature dependencies of the on-chip sensor resistances while collecting data using a reference thermo probe.

### 2.3. Interdigitated Electrode Structure (IDES)

The IDES consisted of two interdigitated electrodes with widths and distances of 50 µm (Figure 1B). The IDES impedance data were recorded between the inner electrode and an engulfing outer electrode, which was grounded to improve the level of electrical shielding.

### 2.4. Multi-Electrode Array (MEA)

**Figure 2:**
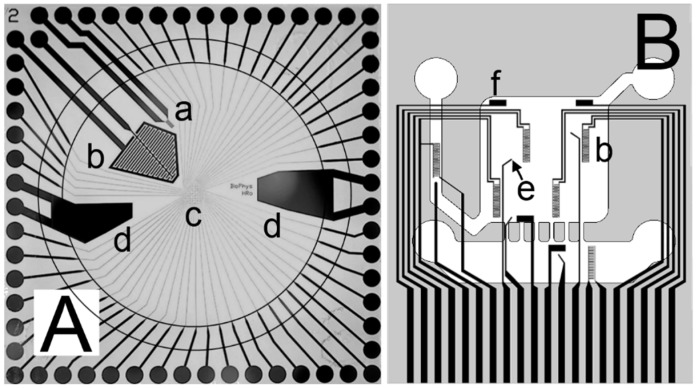
(**A**) Microscopic image of the GNC surface (16 mm × 16 mm) with the integrated sensors: temperature probe (a); IDES (b); MEA (c); stimulating field and ground electrodes (d). An open circular glass trough was glued to the GNC surface (circles indicate the thickness of the trough wall). (**B**) Microscopic image of the glass-metabolic chip surface (22 mm × 27 mm) with five oxygen sensors (e); four pH sensors (f) and 6 IDESs (b). The microfluidic PDMS structure holding the microfluidic volume is light grey. The four circular structures are inlet or outlet connector sites.

The MEA (Figure 1C) was designed to detect the spontaneous electrical activity of cells, e.g., that of neuronal networks exposed to chemical substances. The MEA consisted of 52 microelectrodes and was located in the center of the GNC (Figure 2). The on-chip conducting Pt paths within the cell-culture trough were 5-µm wide, insulated with 1-µm Si_3_N_4_, and terminated in circular MEA pads. For the tests, eight different MEA layouts with rectangular grid patterns were produced using two different inter-MEA-pad distances (100 and 170 µm) and four MEA-pad diameters (15, 20, 25 and 35 µm). The external GNC-contact pads were connected to a home-made 52-channel headstage (preamplifier and filter) via the gold-spring needle contacts of a contact-adapter [10]. The GNC and headstage were fitted into a microscopic stage pick-up. The neuronal signals detected by the MEA were finally fed into a Plexon^®^ amplifier connected to a PC-data card (MHP64 PostAmp, Plexon Inc., Dallas, TX, USA). Plexon^®^ MEA Server software (Plexon Inc., Dallas, TX, USA) allowed recording up to four different action-potential signals traces per electrode in parallel. Data analysis was conducted using the Offline Sorter V.2.8.6 (Plexon Inc., Dallas, TX, USA) and NeuroExplorer V.3.2.66 (Nex Technologies Inc., Littleton, MA, USA) programs.

### 2.5. Stimulation and Ground Electrodes

Two large Pt electrodes were in direct galvanic contact with the cell-culture medium at both sides of the cell-culture area. The two opposing electrodes provided the ground potential, while the spontaneous electrical activity of cells was detected using the MEA. The electrodes could be used to test the possible cell-stimulating effects of electric fields (Figure 1D).

### 2.6. Oxygen (O_2_) Sensor

The amperometric oxygen sensor was operated in the Clark mode, except that a bare-Pt working electrode was used (Figure 1E) [38]. Omitting an oxygen-selective membrane significantly reduced the complexity of producing the chips. The working electrode was circular (diameter 25 µm), with an area of 4.9 × 10^−4^ mm^2^. External Ag/AgCl electrodes (Microelectrode Inc., Bedford, NH, USA), which were used as counter and reference electrodes, were connected to the fluidic outlet. Measurements were conducted using a commercial potentiostat (Palmsens, Palm Instruments BV, BZ Houten, Netherlands). The sensors were two-point calibrated using air-saturated (21% O_2_) and oxygen-free (0% O_2_) cell-culture medium. To produce the oxygen-free reference, the medium was degassed with 1% sodium sulfite.

### 2.7. pH Sensor

A rectangular 60-nm thick Si_3_N_4_ layer with an active area of 1.24 mm × 0.46 mm was used as the pH-sensitive element of the sensor (Figure 1F). Si_3_N_4_ is a cell-culture compatible and robust material. Matsuo et al. [39] first described Si_3_N_4_ as a possible gate material for ion-sensitive field-effect transistors (ISFETs) [40]. The Si_3_N_4_ layers were sputtered onto the Pt structures of new chips using a high-frequency magnetron sputter system (LA 320S, Fa. von Ardenne-Anlagentechnik GmbH, Dresden, Germany). Potentiometric measurements were conducted using a potentiostat (Palmsens, Palm Instruments BV, BZ Houten, The Netherlands). An Ag/AgCl microelectrode (Microelectrode Inc.) in the fluidic outlet of the GC^3^ was used as the reference electrode.

### 2.8. Electro-Poration Electrodes

The size of the electroporation chip was 7 mm × 7 mm. Its disk-shaped 7-µL sample volume was confined by a microscopic cover slip-lid placed above the central circular opening in the passivation layer (Figure 1G). The presence of six parallel electrodes allowed achieving five different electrode distances, i.e., producing five different field strengths using the same electroporation pulse (Figure 1G). Two temperature sensors were used to control the temperature throughout the electroporation experiment. The electroporation pulses were generated by square-topped signals with a key ratio of 1:1 produced using an HP 8130A function generator (Hewlett Packard GmbH, Waldbronn, Germany). The output voltage could be amplified up to 20 V_pp_ at frequencies as high as 60 MHz using a home-made amplifier. An HP 8116A pulse generator (Hewlett Packard GmbH) was used to generate the pulse-length gating signal for the function generator. To produce high-fidelity pulse signals, the signal cables were terminated by 50-Ω resistors located near the electroporation chips.

### 2.9. Dielectrophoresis

Dielectrophoresis, which is the translation of polarizable objects in inhomogeneous AC fields, is based on forces generated by the interaction of the object's polarization charges with the inducing AC field. Depending on the relative polarizabilities of the object and the external medium, positive or negative DEP is observed, i.e., the object is translated toward areas of high or low field intensity, respectively. In homogeneous fields, the same forces may lead to object elongation and compression [50], and the translational forces are nullified due to their symmetry [26]. In this study, we employed inhomogeneous fields around MEA microelectrodes for the positive DEP-mediated allocation of neuronal cells.

### 2.10. Electro-Thermal Micro-Pumps (ETµPs)

In ETµPs, which exploit generalized DEP forces, forces evolve from the field interaction with polarization charges smeared around a heated pump-medium volume. ETµPs were first described by Stubbe et al. [41]. The pump structure shown in Figure 1H consists of two parallel field electrodes and a meandering heating element. The walls of the 120-µm wide pump channel (dark grey area between the electrodes) were composed of a 60-µm high layer of photo-resistant material. The two field electrodes generated a largely homogeneous AC pump field, which acted on the asymmetrically heated pump-medium volume [5].

## 3. Sensorized Glass Chips

### 3.1. Glass Neurochip (GNC)

The GNC (Figure 2A) included an MEA with a temperature probe, an IDES, and ground electrodes. The chip contained an open 5.0-mm deep glass-ring trough (inner diameter: 8 mm) that was glued onto the chip surface using a medical silicone adhesive (Med-1511, NuSil Technology, Sophia Antipolis, France). The trough provided a cultivation area of approximately 50 mm^2^ and could contain a maximum of 250 µL (generally 200 µL was used) of cell culture medium.

### 3.2. Glass Metabolic Chip

The glass metabolic chip (Figure 2B) was a further development of the chip created by Bonk et al. [51]. The microfluidic structure was imprinted in a poly-dimethyl-siloxane (PDMS) layer on the bottom side of a poly-methyl-methacrylate (Plexiglas^®^ GS, Acrylics Ltd. Niederfischbach, Germany) lid containing four microfluidic ports with tube connectors. The lid was attached to the PMMA bottom plate using two screws, which compressed the PDMS structure to achieve a tight seal between the microfluidic structure and the chip. The central sensorized cell-culture volume (light quadratic area in the center of Figure 2B) was 500 µm high and was connected through six 500-µm high channels with a 1-mm high medium reservoir area (bottom structure). The reservoir had an overall surface area of 168 mm^2^ and a total volume of 100 µL. For cell seeding and medium exchange, 0.8-mm diameter syringe needles were embedded in the PDMS structure to form four fluidic ports. In the initial seeding tests, the cells were either seeded in the lower reservoir compartment through the two lower connectors or were directly flushed into the central quadratic cell-culture area. After more than 3 days post-seeding, the four vertical connecting channels between the reservoir-compartment and the quadratic cell-culture volume were perfoliated. This finding suggests that medium exchange between the lower on-chip reservoir-compartment and the external reservoirs reduced the fluidic shear stress for the cells in the culture area. In future setups, ETµPs placed in the vertical connecting channels will allow for a modest level of medium exchange between the reservoir and the cell-culture compartments.

After these tests were completed, cell culturing was conducted with the lower left and top right connectors being used as the inlet and outlet, respectively. The inlet and outlet were connected with Teflon (PTFE) tubes to the medium flask and an external peristaltic pump [51]. The other two connectors were sealed. The sealed system could be operated in the microorganism-reduced environment of a simple heating incubator.

## 4. Cell Culture

MC3T3-E1 and L929 cells were purchased from the Leibniz Institute DSMZ (German Collection of Microorganisms and Cell Cultures), Braunschweig, Germany. MG63 cells were only used in the acidification experiments. For details please see: [51]. PNCs were isolated from the frontal cortex of embryonic (E16) NMRI mice under sterile conditions. The mice were provided by the “Zentrale Versuchstierhaltung” (core facility) of the University of Rostock. Chicken red blood cells were obtained from the BfR (Bundesinsitut für Risikobewertung, Berlin, Germany).

To culture PNCs, as well as the MC3T3-E1 and L929 cell lines, GNCs were coated with 50 µL of poly-d-lysine (PDL) (100 µg/mL) and were incubated overnight at room temperature. Prior to sowing the cells, the chips were coated with 50 µL of laminin/DMEM (1:20; 25 µg/mL) for 2 h. Due to their excellent adhesion abilities, the MC3T3-E1 cells were cultured without adhesion factors. All cell types were allowed to adhere for 4 h at 37 °C with 5% CO_2_ and 95% humidity.

The PNC cell-culture medium consisted of 88% Dulbecco’s modified Eagle’s medium (DMEM) containing phenol red, 10% equine serum, 1% penicillin/streptomycin solution (100 U·mL^−1^ penicillin/100 µg·mL^−1^ streptomycin) and 1% L-glutamine (200 mM). Eighty-nine percent of the cell-culture volumes consisted of either α-medium (MC3T3-E1) or DMEM (L929). Both of these media were supplemented with 10% fetal bovine serum, 1% penicillin/streptomycin solution (100 U·mL^−1^ penicillin/100 µg·mL^−1^ streptomycin), phenol red and sodium bicarbonate.

For the oxygen-consumption and acidification measurements, the media were exchanged using a peristaltic pump (Ismatec IPX-N, IDEX Health & Science GmbH, Wertheim, Germany) every 5 h for more than 24 h. For the acidification measurements, all of media were prepared without sodium bicarbonate. All of the cell-culture media and supplements were purchased from Biochrom AG (Berlin, Germany).

## 5. Results and Discussion

### 5.1. Temperature Sensors

On-chip temperature sensors are important for monitoring the effects of local heat sources, such as on-chip electro-thermal micro-pumps, field electrodes for cell manipulation, heating elements for thermally driven chemical reactions, such as PCR, and for measuring heat dissipation by the local electronic periphery, such as that of the resistors used for high-frequency termination in the electroporation experiments [21,26,41,42,43].

**Figure 3:**
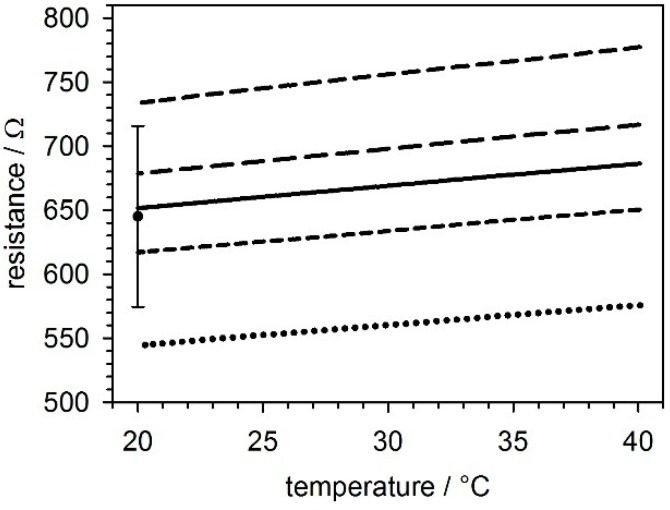
Temperature-dependent resistance values of five temperature sensors in five different GNCs at between 20 and 40 °C, as well as the averaged resistance values and standard deviations at 20 °C. More than 700 data points were obtained for each curve.

The reference temperatures for calibrating the temperature sensors were monitored using an PT-104A thermo-probe placed in the trough medium. The chip temperature was adjusted to 40 °C before the heater was turned off, and the sensor data were recorded in 5-s intervals as the temperature was decreased to 20 °C. The resistances *R* values of the temperature sensors showed a linear dependence on temperature *T* according to the following equation:

(1) $$R=R_{20{}^{\circ}\text{C}}\left(1+\alpha \;\left(T-20{}^{\circ}\text{C}\right)\right)$$

with *R*_20 °C_ and α being the resistance at 20 °C and the temperature coefficient, respectively. Figure 3 shows fits of Equation (1) to the data obtained using five sensors (Figure 1A) as well as their averaged resistance values and standard deviations at 20 °C.

Linear fits of the sensor data obtained using five chips provided a temperature coefficient of α = 0.00283 ± 0.00013 K^−1^ (mean values ± SD) and an *R*_20 °C_ resistance of 645.12 ± 70.63 Ω (Figure 3). All correlation coefficients were better than 0.999. Our probes were slightly less sensitive than were commercial standard probes composed of pure platinum (α = 0.0039 K^−1^ for the commercial Pt1000 industrial standard probe, cf. DIN EN 60751, IEC 60751 or ASTM E-1137).

Applying the rule of proportion to the contributions of the 100-nm thick platinum and the 10-nm thick titanium (α = 0.0026 K^−1^) adhesion layer to the overall layer thickness of 110 nm suggests that our sensors would have a higher level of sensitivity, with a temperature coefficient of α = 0.0035 K^−1^. The reasons for this aberration may be that there were distortions in the metal structures at the glass-titanium, the titanium-platinum, and the platinum-silicon nitride interfaces. Alloys, which may have formed in the metal diffusion regions, are known to have temperature coefficients that are hard to predict.

The high linearity and reproducibility of the sensor resistance values justified an individual two-point calibration of each chip. Despite their deviations from the parameters of the commercial Pt1000 probes (produced using pure platinum with an *R*_20 °C_ of 1000 Ω), the on-chip probes could be directly used with commercial thermostats.

While neuronal network activity was being monitored, the temperature sensors (Figure 1A) of the GNCs (Figure 2a) allowed us to crosscheck the temperatures that were adjusted using the heater of the microscopic stage [10] and the actual temperatures in the medium in the open GNC troughs determined using commercial temperature probes. Precise temperature control is important because neuronal activity is strongly temperature-dependent.

While the electroporation experiments were being conducted, the two temperature sensors of the electroporation chip allowed continuously controlling the temperature. The heat dissipated by the terminating 50-Ω resistors, which were located near the electroporation chips, resulted in maximum temperature increases of up to 1 °C, which peaked at approximately 1 min after the pulse was delivered.

### 5.2. IDES Measurements

**Figure 4:**
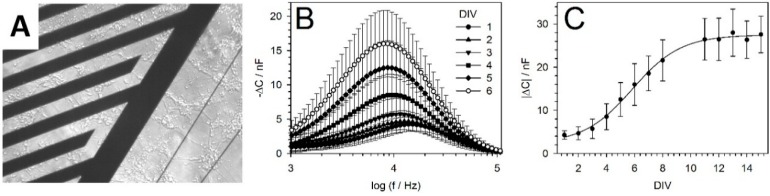
(**A**) Microscopic image of a neuronal PNC network on the IDES of a GNC; (**B**) Frequency dependence of the IDES-capacitance differences (−∆*C*) (with-cells minus control (without cells)) [10,51]. The data obtained using eight GNCs were averaged over six DIV. The measurements revealed the characteristic magnitude and frequency shift of the −∆*C*-peak during cell proliferation; (**C**) Fit of Equation (3) to the absolute values of the −∆*C*-peak magnitudes shown in B.

IDES impedance measurements were conducted using an impedance/gain-phase analyzer (HP4194A, Agilent Technologies, Böblingen, Germany) in the frequency range of 1 to 100 kHz. The impedance data were interpreted assuming the presence of simple resistor-capacitor pair in parallel. The frequency-dependent IDES capacitance C was calculated from the recorded real (*Z_real_*) and imaginary (*Z_imag_*) impedance parts using the following equation:

(2) $$C=\frac{-Z_{imag}}{\omega \;\left(Z_{real}^{2}+Z_{imag}^{2}\right)}$$

with *ω* being the circular frequency. Calibration measurements were performed using the IDES of eight GNCs after the chips were coated according to the cell-culture protocol, although no cells were seeded on them. The IDES capacitance was reduced in the presence of cells and decreased as the number of cells increased. Plotting the –Δ*C*-capacitance obtained from the differences between the cell-covered and cell-free IDES over the frequency yielded spectra with peaks (Figure 4B). A more detailed model showed a largely linear dependence of the peak magnitude on the cell-covered IDES area [10]. Accordingly, daily recordings of the –Δ*C*-peaks reflected cell proliferation and allowed calculating the characteristic cell proliferation rates. For this calculation, a modified logistic Verhulst-Pearl equation was applied to the absolute values of the –Δ*C*-peak magnitudes averaged over eight GNCs as follows:

(3) $$\Delta C(DIV)=\frac{\Delta C_{0}}{\frac{\Delta C_{0}}{\Delta C_{max}}\;+\left(1-\;\frac{\Delta C_{0}}{\Delta C_{max}}\right)\;e^{\frac{\ln\;(2)DIV}{t_{P}}}}$$

with *t_p_* and *DIV* describing the characteristic proliferation rate of the cells and the cultivation period as “days in vitro”, respectively. Δ*C*_0_ and Δ*C_max_* were the plateau values without cells and with maximal cell coverage, respectively.

Figure 4 concerns the proliferation of PNCs for 15 days, i.e., until DIV15. The PNCs were prepared and were seeded on the GNC as described in the "Cell culture" section. The first impedance measurements of the PNCs and cell lines were conducted on DIV1. The PNCs were cultivated until DIV15. All of the data were interpreted using Equations (2) and (3). As examples of the data obtained, Figure 4B,C demonstrate the *t_p_* calculated for PNCs cultivated for 15 days. For a clearer presentation, the *−∆C* spectra shown in Figure 4B concern only DIV1–DIV6. Fitting the data obtained using Equation (3) yielded a *t_p_* of 14.28 ± 0.74 h (Figure 4C).

The DSMZ reported *t_p_* values of 21–24 h and 24–48 h for the L929 and MC3T3-E1 cell lines, respectively. Our fits of the data pertaining to these same cells lines with Equation (3) yielded *t_p_* values of 28.2 ± 0.3 h and 11.5 ± 2.5 h on coated surfaces and 11.6 ± 3.6 h and 8.7 ± 4.6 h on uncoated surfaces (data not shown). We think that our relatively short characteristic proliferation rates reflect the specific culture conditions on our chips, i.e., the surface composition, the adhesion-factor coverage and the cell-seeding number.

### 5.3. MEA: Cell Manipulation and Action-Potential Determination

#### 5.3.1. Dielectrophoretic Cell Positioning

DEP was tested as a method for allocating cells to the MEA pads during cell seeding. DEP forces result from the imbalance of the forces acting on the two hemispheres (or hemiellipsoids) of a cell in an inhomogeneous field. The forces depend on parameters such as field strength, cell volume and shape as well as the balance of the effective electrical properties of the cell and the external medium. This balance depends on frequency [28]. The volume of an ellipsoidal cell with the three semi axes *a*, *b* and *c* is obtained using the following equation:

(4) $$V=\frac{4}{3}\pi \;abc$$

For simplicity, it is generally assumed that cells are polarized by a homogeneous field. An argument for this assumption is the (generally) insignificant difference in field strength over an entire biological cell with respect to the overall field magnitude. For semi axis *a* oriented in field direction *x*, the time-averaged DEP force acting on the cell is obtained using the following equation:

(5) $$\langle {\vec{F}}_{DEP}\rangle =\frac{1}{2}\varepsilon_{0}\varepsilon_{e}\;V\;CMF_{a}^{ℜ}\;\vec{E}\nabla \vec{E}$$

with *ε_0_* and *ε_e_* being the vacuum permittivity and the relative permittivity of the surrounding medium, respectively. ${\vec{E}}_{0}$ and $CMF_{a}^{ℜ}$ represent the external electric field and the real part of the Clausius-Mossotti factor along axis a, respectively. For simplicity, a constant field gradient in *x*-direction can be described as $\vec{E}=E_{0}\left(1+\gamma \;x\right)$, with factor *γ* representing the level of inhomogeneity [26]. For an oriented single cell, DEP translation will be observed in or against the field gradient in *x*-direction. Assuming $\nabla \vec{E}=\gamma \;E_{0}$ for short distances *x*, Equation (5) can further be simplified as follows:

(6) $$\langle {\vec{F}}_{DEP}\rangle =\frac{1}{2}\varepsilon_{0}\varepsilon_{e}\;V\;CMF_{a}^{ℜ}\;\gamma \;E_{0}^{2}$$

The Clausius-Mossotti factor (the frequency-dependent part of the induced dipole moment) along the semi axis *a* is defined as follows:

(7) $$CMF_{a}=\frac{\varepsilon_{i}-\varepsilon_{e}}{\varepsilon_{e}+\left(\varepsilon_{i}-\varepsilon_{e}\right)n_{a}}$$

with *ε_e_* and *ε_i_* being the complex permittivity of the external medium and the effective complex permittivity of the cell, respectively. $n_{a}$ is the geometry-dependent depolarizing factor along semi axis *a* [26]. In the case of spherical cells, a=b=c and $n_{a}=1/3$. Equations (4), (6) and (7) can be simplified accordingly.

Positive or negative DEP is observed, depending on the polarizability of the cell relative to that of the surrounding medium. When cells are higher polarizable than is the external medium they are translated toward regions of higher field strength through positive DEP, whereas they are translated toward regions of lower field strength through negative DEP [24,26]. Both effects can be used to separate or to localize cells on chips. The relative polarizability of a cell and the external medium depends on the field frequency. For example, cells in low-conductivity media generally show negative DEP at below and positive DEP at above the frequency range of capacitive-membrane bridging [5].

**Figure 5:**
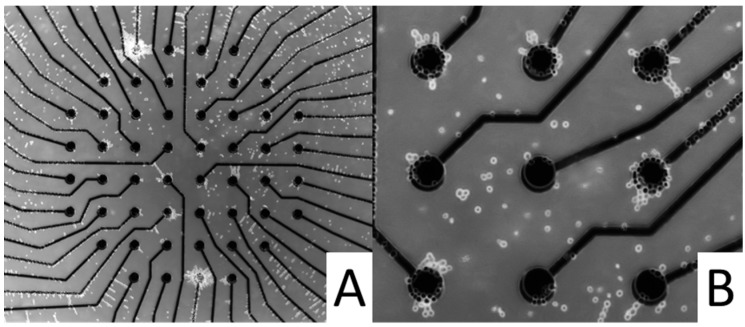
(**A**) Design of the MEA (electrode pad diameter: 35 µm); (**B**) PNCs allocated to the MEA-electrodes in the upper right quadrant through positive DEP (1 MHz, 16 V_pp_).

To increase the probability of detecting neuronal signals from the PNCs after they had formed networks, positive DEP was used to allocate them to the MEA-electrode pads (Figure 2A and Figure 5). In this study, we used MC3T3-E1 cells to demonstrate this principle. To ensure low polarizability of the external medium, the cells were washed twice before they were gently resuspended in a 300 mOsm sucrose solution at a conductivity of approximately 20 mS/m.

For cell allocation, 200 µL of the sucrose/cell suspension was pipetted into the chip. A sinusoidal signal with a frequency of 1 MHz and a voltage of 16 V_pp_ was applied to the MEA-electrodes. Positive DEP toward the MEA pads was observed after all of the MEA-electrodes were energized simultaneously against a wire electrode. After 15 min, most of the cells had adhered to the electrodes (Figure 5B). Some of the cells were allocated along the MEA-pad connectors, which were passivated by an approximately 1-µm thick layer of Si_3_N_4_. Independent tests (electron-microscopic imaging; observation of possible bubbling by driving the MEA electrodes with DC) suggested that the passivation layer was intact. We think that the cells were attracted to the insulated pad connectors via capacitive bridging, possibly in combination with local voltage increases occurring via electrical resonance on the connecting line [25].

Resonance can possibly be avoided by matching the geometric and electrical properties of the connecting lines, passivation layer and driving frequencies. On the other hand, the resonance effect can be exploited for the self-regulation of cell allocation if the empty electrode-pad capacitance and the joint inductance of the external and on chip wiring generated a voltage peak at the MEA pads via electrical resonance, allowing frequency-adjusted cell allocation [52]. Cell collection would then result in a change in the capacitance of a given MEA pad, leading to a reduced resonance-voltage increase and a lower DEP force, which would attract fewer cells to an already-occupied pad. Moreover, this self-regulated voltage decrease would reduce the risk of cell-membrane damage by high field strengths, particularly at the edges of the electrode pads.

#### 5.3.2. Detection of Action Potentials

**Figure 6:**
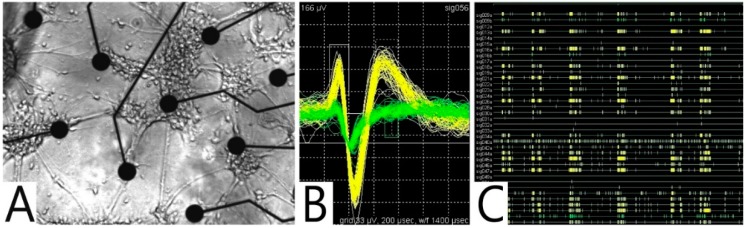
(**A**) Neuronal network of PNCs isolated from the frontal cortex (E16, DIV28). Electrode pad diameter: 35 µm, rectangular pad-center distances: 170 µm; (**B**) Screenshot of the MEA server showing multiple repetitions of the action potential traces of two units detected via the same MEA pad. The peak-to-peak voltages of the large and small signals were approximately 238 and 76 µV, respectively; (**C**) Screenshot of the MEA server showing action potential-spike trains of 37 units that were detected via 34 electrode pads over 20 s.

The spontaneous electrical activity of PNC networks was detected using GNCs. The cell preparation procedures were approved by the local Animal Care Committee and were in accordance with the European Council Directive of November 24, 1986 (86/609/EEC). The brain tissue of mice at embryonic day 16 (E16) was enzymatically digested before suspending and seeding the dissociated cells on coated GNCs [10]. The cells were cultivated for 28 days at 37 °C with 10% CO_2_ and 95% humidity. On the GNCs, the PNCs formed widely ramified neuronal networks that were comparable to the networks formed on culture-flask surfaces (not shown). Figure 6A shows an example of such a network. Figure 6B shows multiple repetitions of two trigger-separated action potential traces, which were detected via the same MEA electrode. Such separated traces, thought to be generated by different neurons, are called units. The complete network exhibited 37 units detected via 34 electrode pads (Figure 6C).

#### 5.3.3. Detection of Action Potentials: Relationship between the MEA-Pad Diameter and the Signal Voltage

The signal voltages of the action potentials detected from a neuronal network depend on the geometry of the MEAs. To determine an optimal geometry, we analyzed the peak-to-peak voltages detected from 40 PNC networks, which were grown on MEAs with the following 8 different layouts: two different inter-MEA-pad distances (100 and 170 µm) in a rectangular grid and four MEA-pad diameters (15, 20, 25 and 35 µm, see Figure 5).

The peak-to-peak voltage of the action potential of each unit was determined using Offline-Sorter software from the peak averages of all of the action potentials within a recorded train of a unit (e.g., those produced within 20 s, as shown in Figure 6C). A software cursor was used to manually determine the average voltage of the peaks (Figure 6B). It must be noted that this method of averaging does not allow distinguishing between two possible causes for the scatter in the peaks of the action potential "bundles" in Figure 6B. The scatter may have been generated by a drift of the baseline, such as that caused by the superposition of a distorting signal, or by actual changes in the action-potential voltages, such as that occurring due to "cellular exhaustion", which may be caused by an increase in the potassium concentration in the external medium occurring during burst activities.

Nevertheless, scatter due to such biological sources was averaged for each MEA pad over longer data-collection periods. We therefore believe that the approach is suitable for determining peak-to-peak voltages and for distinguishing the effects of the MEA-pad geometries on the signal amplitudes.

**Figure 7:**
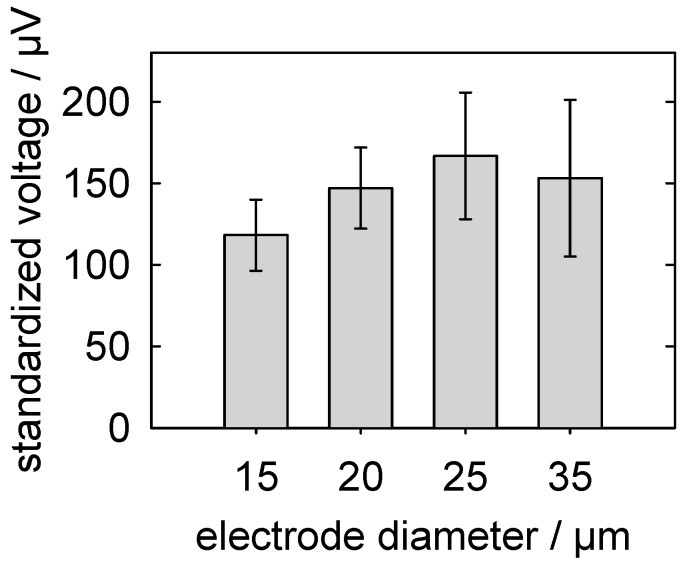
Averaged peak-to-peak voltages (see Figure 6B) of the action potentials of PNC networks that were detected depending on the MEA-pad diameter. The plotted data were obtained using 9 chips (15 µm), 12 chips (20 µm), 10 chips (25 µm) and 9 chips (35 µm). The average number of detected units per chip was 25.

For the first analysis, we used a two-way analysis of variance (ANOVA) by ranks to evaluate whether the inter-MEA-pad distances affected the peak-to-peak voltages (*p* = 0.840). No cross-interference between the diameter and distance was found (*p* = 0.962). The pad diameter was found to have a significant effect on the signal strength (*p* = 0.021) (Figure 7). The peak-to-peak voltages were clearly highest for MEAs with electrode-pad diameters of 25 µm (166.75 ± 38.80 µV). Nevertheless, only the values for MEAs with electrode-pad diameters of 15 and 25 µm were significantly different (*p* < 0.05; all pair-wise multiple comparison procedures, Bonferroni’s *t*-test).

The trends showed that the standard deviations continuously increased with the pad diameter, whereas the peak-to-peak voltages exhibited an optimum curve. The large electrode pads may have reduced the peak-to-peak voltages of the recorded action potentials, which could be attributed to the neuronal structures electrically coupling to only a small area of the pad surface. Accordingly, larger electrode pads would more strongly dampen the signal because of their stronger capacitive coupling to the grounded bulk solution. However, electrode pads with larger diameters cover a wider MEA area and may therefore pick up the signals of more cells, although with different signal strength, resulting in a wider range of scattering (Figure 7).

Our results support our choice of MEAs with 25-µm diameter pads and 100-µm inter-pad distances for routine investigations. Indeed, comparing this geometry with the geometries of commercially available MEA chips showed that our geometry was in the midrange. Commercially, specially designed chips with MEA pad diameters of up to 100 µm and inter-pad distances of up to 700 µm are available and are intended for other electrically active cell types, such as myocardial cells.

#### 5.3.4. Effect of Mefloquine on Neuronal Network Activity

Neuronal networks formed on neurochips are known to respond to chemical substances, changes in temperature or culture conditions, mechanical vibrations, and other factors by changing aspects of their electrical activity, such as the firing rate, action-potential voltage, and bursting behavior. There are abundant reports in the literature describing the results of relevant experiments, such as those performed to investigate the effects of drugs with neurological effects or side effects.

For this study, we chose mefloquine as the neurosensitive drug [53]. Mefloquine (brand-name: Lariam^®^ Roche) was primarily designed as an anti-malaria drug. Nevertheless, it has side effects stemming from its blocking the gap-junction protein connexin, particularly in epithelial, myocardial, neuronal, and retinal cells [53]. This blockage is known to result in a general decrease in the level of neuronal network activity [10].

**Figure 8:**
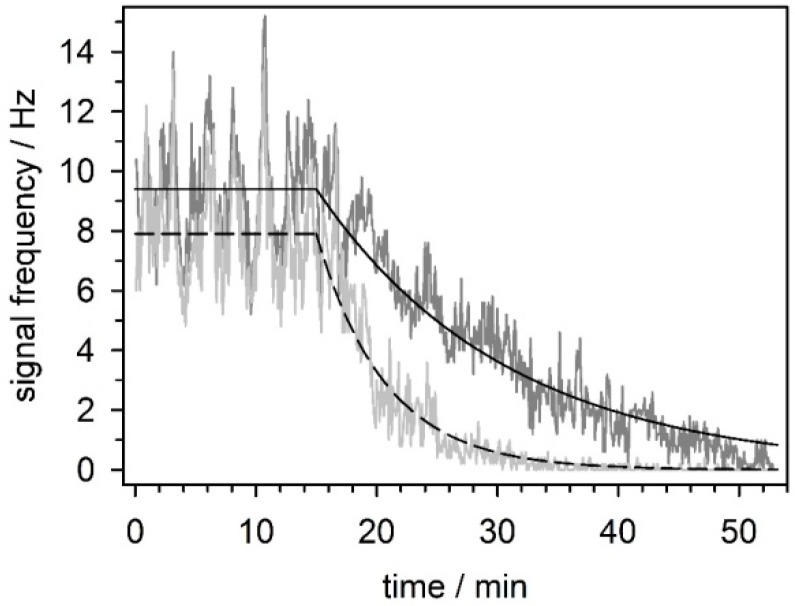
Decrease in the firing frequency of two units of a neuronal network upon exposure to mefloquine. The firing frequencies were averaged (horizontal lines) for 17 min before 10 µM mefloquine was added. The exponential decay functions could be fitted to the mefloquine-induced decrease in firing frequency. The averaged firing frequencies before the addition of mefloquine and the characteristic decay periods were 9.38 Hz and 13.64 min (solid line) and 7.81 Hz and 3.73 min (dashed line), respectively.

PNCs isolated from the frontal cortex were cultured as described above until DIV28. To obtain measurements of the neuronal activity, the activity was averaged for 17 min before 10 µM mefloquine was added, after which it was recorded for another 36 min. The results shown in Figure 8 are an example of the sensitive detection of the effects of a neurotoxic drug on single neurons within a network.

### 5.4. Oxygen Measurements

**Figure 9:**
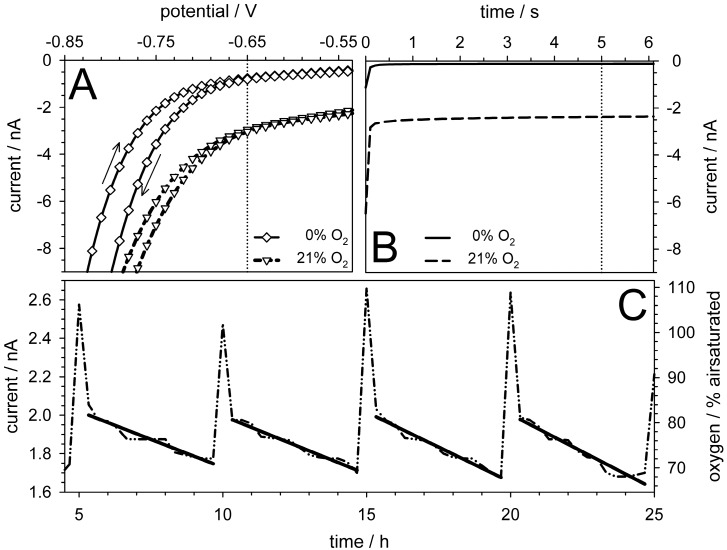
Oxygen-sensor characterization and cell-culture measurements. (**A**) Results of cyclic voltammetry of air-saturated (21% O_2_) and oxygen-free (0% O_2_) media. The vertical dotted line at −650 mV indicates the potential used in the cell-culture measurements; (**B**) Time-dependent current at −650 mV. The current values in the cell cultures were recorded after 5 s after the voltage was applied (vertical dotted line; (**C**) Respiration measurements of MC3T3-E1 cells. The 5 min-long medium exchange performed every 5 h induced current peaks, which were neglected to determine the current-drop rates by linear fitting (tilted bars). Please note the slightly decreasing slopes of the consecutive bars.

The amperometric oxygen sensors (Figure 1E) were tested using air-saturated and oxygen-free medium. The sensors were operated in the Clark mode, although using a bare Pt working electrode (cathode) and an external Ag/AgCl anode in the fluidic outlet. For the first characterization, cyclic voltammetry (−0.9 to +0.5 V, scan speed of 0.1 V/s, step width of 0.01 V) was used to observe the possible effects of the medium and the PDL surface coating and to determine the optimal working potential. To obtain a more stable signal, the current was recorded 5 s after the voltage was applied to the sensor (Figure 9B). The experiments showed the negligible effect of the PDL surface coating on the current at a working potential of −650 mV (Figure 9A). This working potential was chosen for measurements in cell-culture medium (instead of −700 mV, which is used for commercial electrodes) to ensure better discrimination of alterations in the electrode properties caused by electrochemical processes, particularly at less than −700 mV [51]. The sensor was calibrated using a two-point calibration because its current was proportional to the oxygen concentration [38]. Under cell-culture conditions (37 °C), signal currents of −2.35 ± 0.04 nA and −0.08 ± 0.05 nA were detected for air-saturated (21% O_2_) and oxygen-free (0% O_2_) medium, the latter of which was degassed using 1% sodium sulfite. These currents were used as references in the experiments in which MC3T3-E1 cells were cultivated in a microfluidic chip setup comparable to that shown in Figure 2B [51].

The medium was exchanged using a peristaltic pump at a flow rate of 300 µL/min for five min every 5 h during a 24-h period (Figure 9C). The measuring current was recorded every 20 min, and the data points obtained were used to determine the current-drop rates (slopes) by linear fitting. Consecutively increasing current-drop rates of −0.058 ± 0.005, −0.060 ± 0.003, −0.073 ± 0.004, and −0.078 ± 0.006 nA per hour were observed. The increasing current drop rates were caused by the increasing oxygen consumption resulting from cell proliferation.

We found that the absence of an oxygen-permeable Clark membrane in our system did not significantly increase the error rate in the detected oxygen concentrations. One explanation for this finding is that the reduced working potential that was used, facilitated discriminating between oxygen and other redox-active compounds. Another explanation for this finding is the high level of stability of the medium properties (ionic and molecular composition, conductivity, temperature) under the cell-culture conditions.

Numerical simulations showed that sensor zapping reduced the oxygen concentration only within a radius of approximately 100 µm around the sensor during the 5-s measuring periods at 20 min intervals. The interval before the next recording was sufficient for re-equilibration of the oxygen in the system to occur. Simulated oxygen-concentration changes were insignificant beyond a radius of 200 µm.

### 5.5. pH Measurements

Matsuo et al. [39] were the first to describe Si_3_N_4_ as a pH-sensitive gate material for the production of ion-sensitive field-effect transistors (ISFETs) [40]. Si_3_N_4_ is cell culture-compatible and robust. To characterize the pH sensors (Figure 1F), the sensor and external reference electrodes (Ag/AgCl microelectrode, Microelectrode Inc.) were exposed to test solutions (phosphate-buffered saline titrated with HCl or NaOH) in the range of pH 5 to 9.

After an induced pH change, 20, 40, and 60-nm thick layers Si_3_N_4_ reached their voltage plateau values within 120 s, independent of the layer thickness. Further testing was conducted using 60-nm thick sensors due to their higher level of stability under cell-culture conditions (Figure 1F).

**Figure 10:**
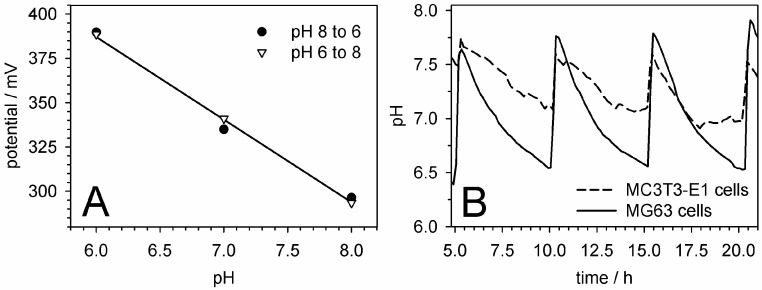
(**A**) Calibration measurements obtained using 60-nm thick Si_3_N_4_ sensors. Linear fitting yielded a mean slope of −44.6 ± 2.1 mV per pH step (*R*^2^ = 0.99), which was independent of the direction of the pH change; (**B**) Acidification behavior of proliferating MC3T3-E1 and MG63 cells cultivated at 37 °C in the microfluidic volume of a glass metabolic chip (Figure 2B). The medium was exchanged over a five-min period every 5 h using a peristaltic medium-exchange pump. Increasingly rapid medium acidification rates were observed upon consecutive medium exchanges.

To determine the sensitivity and response time of the sensors, the pH of the medium was changed in integer steps in the range of pH 5 to 9. After each pH exchange, the sensor potential was monitored using a PalmSens potentiostat (PalmSens BV, Utrecht, The Netherlands). The pH values were recorded after 120 s. The sensors reached approximately 90% of their 120-s signal magnitudes after less than 20 s. The sensor potentials were strictly linear within the pH range of 6 to 8 (Figure 10A), with reproducible sensitivities that were typically greater than 40 mV per pH step, which corresponded to approximately 75% of the idealized Nernst difference of −59 mV at room temperature. This level of sensitivity was comparable to that of ISFETs with Si_3_N_4_ gates [40]. We found no significant differences in the sensitivity level or response time of sensors of various sizes (results not shown).

Before the medium-acidification behavior of cells was analyzed, the sensitivity of the pH electrode in the microfluidic system was calibrated using two pH test-buffer media (Carl Roth GmbH, Karlsruhe, Germany). The pH-electrode potentials were detected relative to that of an Ag/AgCl reference electrode at 37 °C. After rinsing the system, 1.5 × 10^6^ MC3T3-E1 or MG63 cells per mL were seeded into a system in which the chip surface was not pre-coated. The cells were allowed to adhere for 4 h. To obtain the data shown in Figure 10B, measurements were taken after an initial 5-min pump cycle at 0 h. The flow rate of the peristaltic pump (Ismatec IPC-N, Cole-Parmer GmbH, Wertheim, Germany) was adjusted to 150 µL/min to fully exchange the medium in the system (volume: approximately 100 µL) and the tubes within five min. Between the four pump cycles, potential changes of 45.1, 48.7, 50.6 mV (MG63) and 30.5, 26.18, 34.3 mV (MC3T3-E1) were observed, corresponding to shifts of 1.14, 1.23, 1.28 (MG63) and 0.64, 0.55, 0.72 (MC3T3-E1) pH units, respectively.

The pH shifts caused by cellular metabolism were significantly greater than the drift of −0.45 mV/h in the sensor potentials observed in the cell-free control experiments, corresponding to approximately 0.019 pH unit/h. Nevertheless, despite the drift in the absolute potentials that was detected (compared to the increasing peak magnitudes shown in Figure 10B), the sensitivity (potential difference per pH step) of the sensors was largely stable for monitoring periods much longer than 24 h. This finding suggests that the drift could be accommodated by referencing the sensor voltage at the end of the pump cycle with the pH of fresh medium. Additional tests are required to determine the stability of the Si_3_N_4_-sensor layers when they are subjected to cleaning and sterilization procedures and to long-term use under cell-culture conditions [10,13].

## 6. Electro-Thermal Micro Pumps (ETµPs)

The ETµPs have a simple design. They consist of two passivated or non-passivated field electrodes located in a straight microfluidic channel with an asymmetrically located heating element (Figure 2B) [42]. The asymmetric location introduces the symmetry break required for generating the pumping effect. Alternative designs, in which the symmetry break is introduced in the channel geometry, do not require a heating element [42]. Both designs take advantage of the temperature-dependent polarizability of the medium. In ETµPs with a biased thermal gradient in the range of a homogeneous AC electrical field, the pumping media are asymmetrically polarized [41,42,43]. The interaction of the asymmetrically induced, smeared polarization charges with the inducing homogeneous field generates volume forces in the pumping medium. Accordingly, ETµPs can pump all media with a temperature-dependent polarizability. For aqueous media, the pumping direction is reversed at frequencies below and above the Maxwell-Wagner frequency, i.e., at frequencies at which conductivity or permittivity effects dominate the polarizability of the medium [5]. In contrast to electro-osmotic pumps, ETµPs are operated at frequencies at which electrolytic processes and electrode deterioration are avoided. Passivation layers at the electrode surfaces are capacitively bridged at the operating frequencies of ETµPs. Passivation prevents the galvanic contact of the metal electrodes with the pumping fluid, thus preventing the occurrence of electrolytic, biochemical and cell-physiological processes. Moreover, the capacitance of the passivation layer can play a role in the resonance circuit, enhancing the pumping efficiency [44].

**Figure 11:**
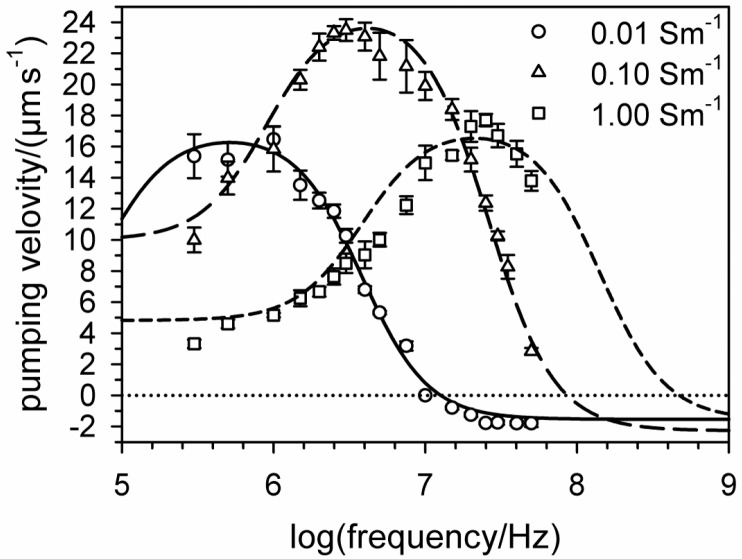
Comparison of the pumping velocities measured using a passivated ETµP as shown in Figure 1A for media with conductivities of 0.01, 0.1 and 1.0 Sm^−1^ (symbols: mean values ± SD of four measurements each) and the corresponding theoretical curves. The electrode voltage and the heating power were 20 V_rms_ and 120 mW, respectively.

For aqueous pumping media, the possible conductivities range from extremely low to greater than physiological values (Figure 11). The simple construction and small size of the ETµPs makes them highly suitable for integration into the microfluidic structures of CMMSs. The pumps can be used to generate flow in the cell culture medium to supply nutrients to the cells or to distribute small amounts of drugs, substances or toxins within the microfluidic cell-culture volumes.

The pumping and electrical properties of ETµPs can be predicted using 3D finite-element models and equivalent-circuit diagrams [44], which facilitates designing new pumps and reduces costs. For example, we found that pumping was more efficient in smaller structures than in pumps in the 100-µm size range. The explanation for this phenomenon is that temperature distribution is determined solely by thermal conductance in small structures, which prevents the disruptive effect of pump flow on the temperature distribution in the pump medium. In larger structures, pump flow and convection can distort the temperature distribution and reduce the pumping efficiency. With their wide operational frequency range and simple structure, ETµPs overcome several drawbacks of electro-osmotic pumps or traveling-wave pumps [5,42].

## 7. Electro-Poration Electrodes

**Figure 12:**
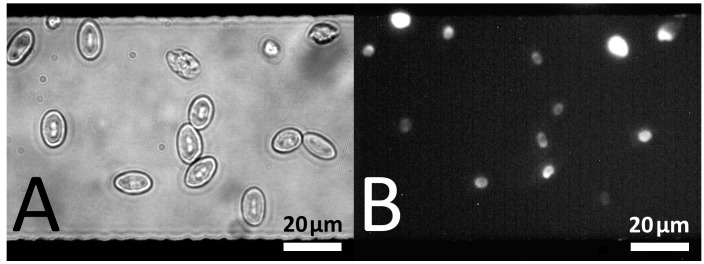
Microscopic images of differently oriented chicken red blood cells suspended in propidium iodide-containing medium before (**A**) phase contrast and (**B**) fluorescence after electro-permeabilization. The cells were exposed to a 10-ms 10-kHz AC pulse of 16 V_PP_, corresponding to 200 kV·m^−1^, which passed between two parallel electrodes separated by 80 µm. Only cells with permeabilized membranes are penetrated by propidium iodide, which can be detected by fluorescence microscopy after it has bound to DNA or RNA.

The electrical induction of membrane pores has been discussed since the late 1970s [54], before it was shown that small molecules, such as dyes, ions or drugs, can pass through field-induced pores [55,56,57]. The efficiency of electropermeabilization depends on several electrical parameters, including the medium conductivity [58], pulse shape and duration [59], field frequency, field strength, and field properties, such as the linear or rotating orientations [60]. Additionally, cell size, shape and orientation, and temperature greatly affect the process [59]. The parameter dependencies can be studied in detail using artificial membranes. Electropermeabilization of cells can be visualized by staining the cytoplasm with fluorescence dyes as shown in Figure 12 [61,62].

Electrically, suspended cells can be modeled as ellipsoidal objects consisting of a non-conducting membrane confined by aqueous media. The membranes of cells exposed to sinusoidal AC fields exhibit a sinusoidal polarization with a phase shift, which depends on the characteristic time constant of membrane charging [5]. For oriented spheroidal (ellipsoids of rotation) cells with the principal axes *a* = *b* and *c*, the maximum induced potential at frequencies below the characteristic time constant of membrane charging can be expressed as follows:

(8) $$\Delta \varphi_{a}=\left(\frac{a+2c}{a+c}\right)\;aE\text{ and }\Delta \varphi_{c}=\left(\frac{a+2c}{2}\right)\;E$$

with axis *a* or *c* being oriented in the field direction [27,63].

With increased induced transmembrane potentials, reversible and irreversible dielectric membrane breakdown, i.e., poration of the lipid membrane matrix may occur [63,64]. The conductivity of transiently induced aqueous pores in the case of reversible membrane breakdown depends on the membrane voltage [65,66]. Under certain conditions, extracellular molecules that do not normally penetrate biological membranes can be introduced into a cell. An example of such a case is the electrotransfection of cells by foreign DNA [49,67]. Reversible breakdown can also be applied to produce hybrid cells via cell fusion [68], such as those used for the production of monoclonal antibodies [49]. In more recent developments, electro-poration of tissues has been used for electro-chemotherapeutic cancer treatment, gene therapy and transdermal drug delivery [46,47,48,69,70,71,72].

Although the average pore size is stable at subcritical field strengths, the pore radius increases at supercritical field strengths, leading to membrane rupture after a critical pore size is reached, resulting in cell lysis and death.

Microfluidic systems have been developed for the electrotransfection and hybridization of cells as well as for cell lysis prior to genetic analysis [73,74]. In the future, integrating specific, highly sensitive electrodes into the system will allow the rapid detection of specific DNA or RNA sequences, such as those of contagious microorganisms [21,22].

## 8. Conclusions

The European REACH (registration, evaluation and authorization of chemicals) policy has been in effect since 2007 [75]. This policy presents a challenge for the producers of chemical and pharmaceutical substances because it requires determining the possible adverse effects of more than 30,000 substances. Moreover, the European and American test guidelines require investigations of the possible neurotoxic and developmentally neurotoxic effects of substances [15,16,18,19]. This policy will clearly increase the demand for methods that replace animal experimentation and broaden the market for diagnostic devices that can evaluate toxic effects at the cellular levels. Non-invasive, multi-parametric online monitoring of the physiological parameters of cultured cell may be a valid replacement for animal experimentation.

We believe that sensorized GC^3^s are a versatile tool for this approach due to their favorable cell-culture properties and robustness. All of the sensors and actuators presented here were produced using a small number of standard wafer-technological processing steps on the same glass substrate. Depending on the problem being investigated, different on-chip sensors can be integrated with open cell-culture troughs or confined microfluidic systems. The greater robustness and re-usability of a glass substrate compared with that of a silicon substrate will reduce costs and increase the probability of the commercial application of GC^3^s. In addition to their high level of biocompatibility, glass substrates allow the use of a combination of optical sensors, enhancing the number of detectable parameters and allowing the use of various optical microscopic methods, such as staining and fluorescence techniques. Based on our experience, sensorized glass-based chips outperformed their silicon counterparts in cell-culture applications [13]. Multiparametric GC^3^s designed to suit certain cell types and parameters are expected to replace test animals, particularly for pre-screening toxic substances. Their future use will motivate the development of new microsensors and promote the consideration of newly accessible parameters in cell biological models. However, a more detailed understanding of biochemical and cell physiological mechanisms requires monitoring ADME (absorption, distribution, metabolism, and excretion) processes [76]. The development of additional sensors for specific cellular markers or released metabolites will facilitate achieving this goal, particularly when the interplay of multiple cell types, resembling the complex, time-dependent biotransformative processes occurring in mammalian organisms, requires monitoring. The combination with flexible PDMS fluidic structures permits the co-culture of multiple cell lines, the physical stimulation of cells, e.g., osteoblasts, or 3D-scaffolding [51,77]. Microfluidic medium exchange will be essential for co-culturing different cell types or even organoids in separate on-chip culture volumes. In principle, the 2D-limitations of planar cell-culturing systems can be overcome by sandwiching PDMS and chip layers with vertical microfluidic channels. For such systems, integrated sensors and ETμPs will be essential because stacking will reduce the microscopic observability and introduce diffusion limitations in the exchange of compounds.

Monitoring the dynamic behavior of complex cellular systems will be a significant advance over end-point evaluation, which is the method commonly used in toxicology [15,16,18,19,78]. End-point evaluation is of limited use in evaluating hormetic effects, such as the upregulation of certain physiological parameters of cells upon their exposure to low doses of harmful chemical compounds [13,79]. Evaluating cellular hormetic effects in systems biology models might allow identifying cellular "control" parameters, which are the parameters that cells must maintain as stable when under physiological stress.

The existing sensors can detect only integrative parameters in in vitro systems, such as the pH, oxygen level, and the levels of certain substances, such as lactate, and purely physical parameters, such as electric impedances. The integrative nature of these parameters indicates foreseeable problems in the future for replacing test animals with cellular micro systems. Therefore, a future goal of systems biology should be developing models for the interpretation of accessible, integrative sensor data. Models for evaluating the metabolic behavior of networks of specific types of cells, such as the apoptosis of liver cells, are a first step in this direction [80].

## Abbreviations

The following abbreviations are used in this manuscript:

AC: Alternating current
ANOVA: Analysis of variance
CMF: Clausius-Mossotti factor
CMMS: Cell monitoring and manipulation systems
DC: Direct current
DEP: Dielectrophoresis
DIV: Days in vitro
DMEM: Dulbeccco’s modified Eagle’s medium
DSMZ: German Collection of Microorganisms and Cell Cultures
E16: Embryonic day 16
ETµP: Electro-thermal micro-pumps
GC^3^: Glass cell-culture chip
GNC: Glass-neuro chip
IDES: Interdigitated electrode structure
ISFET: Ion-sensitive field-effect transistor
MEA: Multi-electrode array
PCR: Polymerase chain reaction
PDL: Poly-d-lysine
PDMS: Poly-dimethyl-siloxane
PNC: Murine embryonic primary neuronal cells
Pt: Platinum
SD: Standard deviation
SEM: Scanning electron microscopy
Si_3_N_4_: Silicon nitride
